# Timing of cranioplasty after decompressive craniectomy and neurological recovery: A systematic review and meta-analysis

**DOI:** 10.1007/s10143-026-04361-3

**Published:** 2026-06-09

**Authors:** Jorge Alberto Roa Castro, David Felipe Alfonso-Cedeño, Leonardo B. O. Brenner, Lucca B. Palavani, Raphael Bertani, Edgar Ordoñez-Rubiano

**Affiliations:** 1 Faculty of Medicine, Universidad Nacional de Colombia, Bogotá, Colombia; 2 Weldon School of Biomedical Engineering, Department of Biomedical Engineering, Purdue University, West Lafayette, IN USA; 3 Department of Neurosurgery, Houston Methodist Hospital, Houston, TX USA; 4 Department of Medicine, State University of Ponta Grossa, Ponta Grossa, PR Brazil; 5 Max Planck University Center, São Paulo, Indaiatuba Brazil; 6 Hospital Israelita Albert Einstein, São Paulo, SP Brazil; 7 Department of Neurosurgery, Hospital Universitario Fundación Santa Fe de Bogotá, Bogotá, Colombia; 8 Department of Neurosurgery, Hospital de San José – Fundación Universitaria de Ciencias de la Salud, Bogotá, Colombia

**Keywords:** Functional recovery, Cranioplasty timing, Decompressive craniectomy, Neurological outcomes, Traumatic brain injury

## Abstract

Decompressive craniectomy (DC) creates a large skull defect that may alter cerebral physiology and impair neurological recovery. Cranioplasty (CP) may reverse these effects and enhance functional and cognitive outcomes; however, the optimal timing remains uncertain. Prior systematic reviews have been limited in scope and have not fully examined cognitive outcomes, ultra-early CP (< 45 days), or traumatic brain injury (TBI)–specific effects. We conducted anupdated systematic review and meta-analysis following PRISMA 2020 guidelines, prospectively registered in PROSPERO (CRD420251130762). MEDLINE, Embase, and LILACS were searched. Eligible studies included adults undergoing CP after DC with extractable timing comparisons (early ≤ 90 days vs. late > 90 days). Functional and cognitive outcomes were assessed using validated instruments. Two reviewers independently performed study selection, data extraction, and quality assessment (Newcastle–Ottawa Scale). Statistical analyses were conducted in R (metafor). Twenty-one studies (1682 patients; 691 early, 991 late) were included. CP, regardless of timing, was associated with significant neurological improvement. In post-CP analyses, early CP demonstrated significantly better outcomes across several functional scales, including BI, FIM, and KPS, and across pooled functional scores (SMD = 0.52 [0.21–0.83], I² = 87.2%). Cognitive recovery assessed by MMSE also favored early CP (SMD = 0.57 [0.34–0.79], I² = 0%). In TBI-only analyses, the effect remained significant (SMD = 0.74 [0.32–1.15], I² = 88.5%). Ultra-early CP showed a favorable but non-significant trend. Heterogeneity was substantial across analyses. Cranioplasty after DC is associated with significant functional and cognitive improvement. Early cranioplasty (≤ 3 months) is associated with better neurological outcomes than delayed reconstruction, with consistent effects across functional scales and in TBI populations. While these findings are biologically plausible, causality remains uncertain as evidence remains largely observational and with substantial heterogeneity. Well-designed multicenter randomized trials are needed to define optimal timing and strengthen clinical guidance.

**Supplementary Information:**

The online version contains supplementary material available at 10.1007/s10143-026-04361-3.

## Introduction

Decompressive craniectomy (DC) is a common neurosurgical procedure performed to treat refractory intracranial hypertension and improve outcomes in conditions such as traumatic brain injury (TBI) [17, 23], malignant cerebral infarction [21, 54], spontaneous intracerebral hemorrhage [3], and others. The secondary large skull defect after a DC alters cerebral physiology in several ways, changing the transmission of atmospheric pressure, the cerebrospinal fluid (CSF) circulation, and cerebral blood flow, potentially impairing neuronal function and metabolism [13, 46, 51].

Consequently, patients commonly undergo cranioplasty (CP) for cranial reconstruction and neurological rehabilitation, as it may improve functional and cognitive outcomes. While early CP might enhance recovery, optimal CP timing remains unclear [18]. To date, only a couple systematic reviews and meta-analyses have examined this topic, confirming that CP is associated with significant and measurable neurological improvement and suggesting that earlier intervention may yield even greater benefits [10, 31]. However, given the expanding body of evidence, an updated synthesis of the literature is warranted to better inform clinical decision-making.

The present study aimed to update and expand upon existing evidence by analyzing a larger pooled cohort to evaluate the overall effect of CP after DC on functional recovery, irrespective of timing, and the difference in effect between early (≤ 90 days) and late (> 90 days) CP. In addition, this study sought to examine these effects as assessed by higher cognitive function measurement tools, and to explore the potential benefits of ultra-early CP (< 45 days), and whether these effects differ among patients who underwent DC for TBI.

## Methods

This study adhered to the Preferred Reporting Items for Systematic Reviews and Meta-Analysis (PRISMA) statement guidelines [39]. The study protocol was prospectively registered in PROSPERO (ID: CRD420251130762) on August 20, 2025.

### Eligibility criteria

Studies were eligible for inclusion if they included adult patients (≥ 18 years) undergoing cranioplasty following decompressive craniectomy (DC) for any indication, reported a comparison between early and late cranioplasty groups defined according to study-specific thresholds or derivable from reported timing data, provided sufficient quantitative data to enable extraction or reconstruction of outcome measures for both groups, and included a minimum sample size of 10 patients. Eligible study designs included randomized controlled trials, prospective and retrospective observational studies, and case series meeting these criteria. Studies were excluded if they utilized a timing threshold of 180 days or more, included a predominantly pediatric population (> 20% of patients < 18 years) without separable adult data, included more than 20% of patients who did not undergo decompressive craniectomy unless outcomes for DC patients could be isolated, lacked sufficient data to enable comparison between early and late cranioplasty groups, or were case reports, conference abstracts without full text, study protocols, or single-arm studies without a comparator group. No restrictions were applied regarding publication date or language, and neither DC location (bifrontal, unilateral, or bilateral hemicraniectomy) nor implant material (autologous or synthetic) were used as exclusion criteria.

### Search strategy and study selection

We conducted our search on MEDLINE (through PubMed), Embase, and LILACS (through Virtual Health Library) on August 21, 2025. No date or language filter was set. Search terms combined subject headings and free-text keywords for cranioplasty, timing, and neurological recovery. Reference lists of included studies and prior systematic reviews were also screened. Two reviewers (J.A.R.C. and D.F.A.C.) independently screened titles, abstracts, and full texts using predefined inclusion criteria, with disagreements resolved by consensus. Reasons for exclusion at the full-text stage were documented.

### Data extraction and outcome definition

Two reviewers independently extracted data using a standardized template, including study characteristics, sample size, patient demographics, indication for DC, interval between craniectomy and CP, timing classification, and functional or cognitive outcome measures at each assessment point. When information was incomplete, authors were contacted for clarification or supplementary data. If unavailable, data from prior meta-analyses were used. The primary comparison was between early CP (≤ 90 days after DC) and late CP (> 90 days), with the secondary analysis assessing ultra-early CP (≤ 45 days) [18]. Studies utilizing timing thresholds of > 180 days were excluded at this stage. The outcome of interest was neurological recovery, evaluated using standardized scales categorized into functional and cognitive groups. Functional scales included modified Barthel Index (mBI), Functional Independence Measure (FIM), Karnofsky Performance Status (KPS), Glasgow Outcome Scale (GOS), modified Rankin Scale (mRS), and the National Institutes of Health Stroke Scale (NIHSS), while the cognitive group included the Mini-Mental State Examination(MMSE), Montreal Cognitive Assessment (MoCA) and the cognitive component of FIM. This grouping approach followed recent efforts of outcome reporting standardization [33].

### Quality assessment

Study quality was independently assessed by two reviewers using the Newcastle–Ottawa Scale for observational studies, evaluating selection, comparability, and outcome domains [11]. Discrepancies were resolved through discussion.

### Statistical analysis

All analyses were performed using R 4.4.3 with the *metafor* package. Effect sizes were expressed as standardized mean differences (SMD) comparing early and late cranioplasty groups, as well as ultra-early and later groups. For studies lacking change-score variances, the standard deviation (SD) of change was imputed based on pre- and post-cranioplasty SDs using scale-specific within-subject correlations derived from studies with complete data. All outcome measures were transformed, when necessary, such that higher values consistently reflected better neurological function prior to pooling; accordingly, mRS and NIHSS scores were inverted before analysis. Finally, GOSE scores were collapsed to GOS for comparability. For the analysis of cranioplasty effects irrespective of timing, means and standard deviations from the early and late groups were pooled as independent samples. Standardized mean change using change-score standardization (SMCC) was applied to preserve the paired structure of the pre–post data while allowing comparison across studies [12]. Random-effects models (REML) were applied to account for expected heterogeneity. Between-study heterogeneity was quantified using Cochran’s Q, I², and τ². Publication bias was assessed using funnel plot inspection and Egger’s test only for outcome-specific meta-analyses with at least 10 studies [50]. No formal assessment was performed for pooled analyses combining different functional outcome measures or for outcome-specific analyses with fewer studies. Separate pooled estimates were calculated for pre-, post-, and change-score comparisons, with subgroup analyses by outcome scale and TBI-only indication.

## Results

### Selection process

**Fig. 1:**
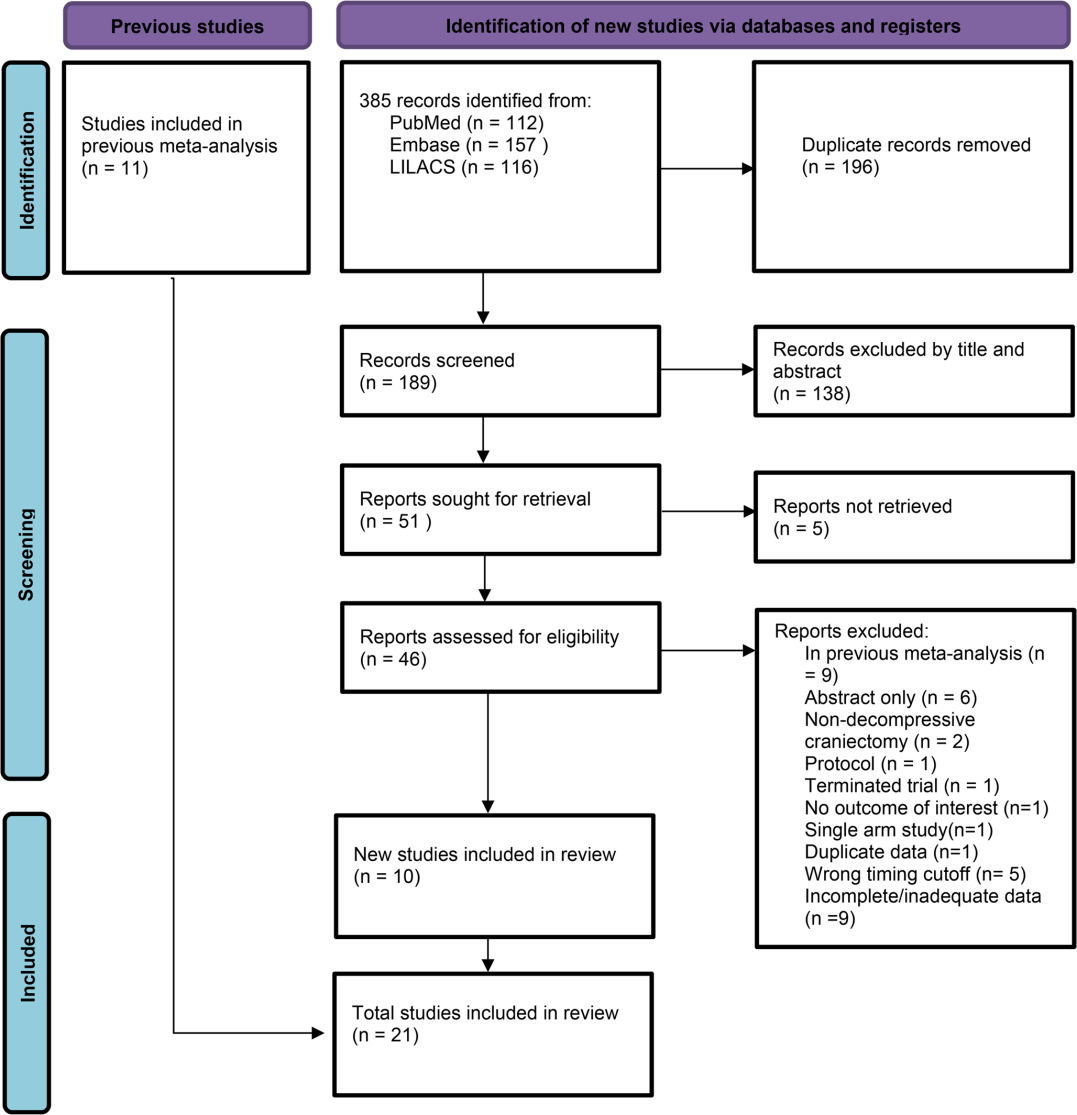
PRISMA flow diagram

Results of the selection process are shown in the PRISMA flow diagram (Fig. 1). 189 non-duplicate articles were screened. 46 entries were retrieved for full-text screening. 11 studies had been previously included in earlier meta-analyses [10, 31], of which 9 studies were also identified through our database search. 8 reports were of inadequate study type. Full articles were excluded due to duplicate data, single arm design, substantial proportion of non-decompressive craniectomy, and lack of post-cranioplasty assessment. Eight studies met inclusion criteria with the reported data [1, 25, 28, 38, 47, 53, 61, 65], and 16 authors were contacted for additional information that would allow for inclusion [2, 5, 9, 20, 24, 27, 36, 43–46, 52, 55, 56, 60, 64]. Only two [43, 55] were able to provide such information, and the remaining were excluded due to wrong timing cutoff or otherwise inadequately reported data. Thus, ten new studies contributing 1094 additional patients were included in this review.

Authors of studies included in previous meta-analyses, but which required exchanging correspondence for inclusion, were also contacted to obtain primary data [4, 14, 16, 19, 41, 48]. However, only one [41] was able to provide such information. Thus, data for these studies was obtained wholly or in part from that reported in previous reviews. One study reported relevant data in medians and inter-quartile ranges [25]; conversion to means and SDs was done using published methods [30, 57]. Finally, three newly included studies did not report SDs of the change in scores [25, 28, 38]. This was imputed by calculating the empirical within-subject correlation of each scale, pooled from the studies that did have the SD of change available. The means of the change in scores were obtained simply by taking the difference in the pre- and post-CP means.

### Newcastle Ottawa scale assessment

Study quality ranged from 5 to 9 (Table 1). Since a between-group comparison was obtained from all studies, they were all considered as observational cohort studies. Only two reported cohort matching [63, 65]. 6 studies did not report pre-cranioplasty scores [1, 7, 16, 55, 61, 65], and three had considerably short follow-up times (< 1 week) [1, 14, 41].

**Table 1:** Description of included studies

Study	Type	Risk of Bias	DC indication	Location	Timing Cutoff	Follow-up	Early *N*	Early Age	Early Males	Early DC-PC interval	Late *N*	Late Age	Late Males	Late DC-PC interval	Outcome scales
Kuo et al., 2004 [26]	Case series	7	TBI 10 (77%), IS (15%), ICH (8%)	NR	90	12.5 ± 2.8 days	7	45.4 ± 11.4	5	59.4 ± 22.44	6	47 ± 19.4	3	195.6 ± 107.56	BI
Zhang et al., 2010 [63]	Prospective	8	TBI	Unilateral	90	1 month BI, 6 months KPS	23	38.7 ± 12.6	15	NR	47	40.1 ± 13.4	31	NR	BI, KPS ^**b**^
Cho et al., 2011 [7]	Prospective	6	TBI	NR	45	1 month	15	51.4 ± 14.9	13	35.2 ± 3.76	21	52.52 ± 15.52	15	62.95 ± 14.82	BI
Bender et al., 2013 [4]	Retrospective	8	TBI (46%), IS (28%), ICH (10%), SAH (14%), SDH (2%)	Bifrontal, unilateral	86	161.7 ± 68.3 days	75	46.6 ± 16.9	NA	62.9 ± 12.9	72	50.1 ± 16.7	NA	111 ± 21	BI, FIM, CRS ^**a, c**^
Huang et al., 2013 [16]	Case series	6	TBI	Bifrontal, unilateral, bilateral	90	> 6 months	76	NA	NA	NA	29	NA	NA	NA	GOS ^**c**^
Paredes et al., 2015 [41]	Prospective	7	TBI (51%), ICH (22%), IS 6 (11%), infection (13%), reabsorption (3%)	Bifrontal, unilateral	90	< 3 days	11	38.3 ± 18.7	7	54.6 ± 21.3	44	44 ± 14.5	30	373.6 ± 21.3	BI, GOS, NIHSS
Cong et al., 2016 [8]	Retrospective	7	TBI	Unilateral	90	1 week, 1 month	22	40.2 ± 11.5	16	80.5 ± 27.6	55	41.8 ± 12.4	40	186 ± 96.6	KPS ^**b**^
Honeybul et al., 2016 [14]	Prospective	6	TBI (72%), IS (6%), ICH (8%), SAH (8%), tumor (4%), infection (2%)	Bifrontal, unilateral	90	< 3 days	20	45.5 ± 16.6	16	64.0 ± 15.2	28	37.2 ± 16.0	22	157.0 ± 125.5	FIM, COGNISTAT ^**c**^
Songara et al., 2016 [48]	Prospective	6	TBI	Unilateral	90	1 week, 1 month	6	34.5 ± 14.6	4	63.7 ± 16.4	10	38.7 ± 12.0	10	195.8 ± 104.9	GOS, GCS, MMSE ^**a, b**^
Kim et al., 2017 [22]	Retrospective	5	TBI (50%), non-TBI (50%)	NR	90	1 month	12	58.7 ± 15.5	7	74.0 ± 14.5	12	51.4 ± 13.1	8	219.0 ± 13.1	FIM, FIMCOG
Jasey et al., 2017 [19]	Retrospective	8	TBI (69%), non-TBI (31%)	Unilateral, bilateral	90	NA	5	40.8 ± 17.8	3	75.4 ± 19.4	8	45.5 ± 19.6	6	135.5 ± 33.7	FIM ^**c**^
Yang et al., 2018 [61]	Retrospective	7	TBI	Unilateral	35	6 months	15	NA	NA	NA	144	NA	NA	NA	GOS
Zhu et al., 2018 [65]	Retrospective	7	TBI	Bilateral	90	6 months	97	47.2 ± 10.6	64	NA	100	43.6 ± 9.8	61	NA	GOS
Kumar et al., 2018 [25]	Prospective	7	TBI	Unilateral	60	1,3,6 months	21	NA	NA	39 (21–28)	21	NA	NA	80 (62–150)	GOSE, MMSE ^**d**^
Ouyang et al., 2020 [38]	Retrospective	7	TBI	Unilateral	90	6 months	48	41 ± 9.9	34	NA	58	40.7 ± 9.6	37	NA	GOS, mRS, NIHSS, MMSE ^**d**^
Aloraidi et al., 2021 [1]	Retrospective	5	TBI (63%), IS (37%)	Bifrontal, unilateral	90	< 1 week	41	32 ± 14.9	NA	NA	60	31 ± 13.3	NA	NA	GOS, mRS
Patel et al., 2023 [43]	Retrospective	8	ICH (29.4%), SAH (11.8%), IS (32.8%), contusion (2.5%), SDH (22.7%)	Unilateral	90	> 6 months	33	59.5 ± 18.9	18	32.2 ± 22.4	86	48.7 ± 15.9	43	238.8 ± 187.7	mRS
Tomar et al., 2024 [53]	Retrospective	7	TBI (68%), IS (32%)	Unilateral	90	1 month	16	49.5 ± 10.9	9	NA	31	53.1 ± 11.5	15	NA	FIM
Sharma et al., 2024 [47]	Prospective	7	TBI (55%), IS (28%), SVT (10%), ICH (7%)	Bilateral, unilateral	90	3 months	30	NA	40	NA	30	38.53	NA	NA	GOS, MMSE
Li et al., 2024 [28]	Retrospective	7	TBI	Unilateral	90	1 month	45	42.6 ± 2.6	26	NA	45	33.2 ± 3.1	24	NA	FIM, NIHSS, MMSE, NCSE ^**d**^
Vreeburg et al., 2024 [55]	Prospective	8	TBI	Bifrontal, unilateral	90	12 months	73	43.2 ± 17.2	51	31.3 ± 17.4	100	41.5 ± 19	72	159.9 ± 86.8	GOS

^a^ Descriptive data taken from previous meta-analyses

^b^ Change scores standard deviations taken from previous meta-analyses

^c^ Full outcome score data taken from previous meta-analyses

^d^ Standard deviations of score changed imputed from pooled per-scale correlation from the other studies

*Abbreviations*: *TBI* traumatic brain injury, *IS* Ischemic stroke, *ICH* Intracerebral hemorrhage, *SAH* Subarachnoid hemorrhage, *SDH* Subdural hematoma, *SVT* Sinus venous thrombosis, *BI* Barthel index, *KPS* Karnofsky Performance scale, *GOS* Glasgow Outcome scale, *FIM* Functional independence measure, *CRS* Coma recovery scale, *MMSE* Mini-mental state examination, *NIHSS* National Institutes of Health Stroke scale, *mRS* Modified rankin scale

### Baseline characteristics of included patients

A total of 21 studies involving 1682 patients (691 early, 991 late) were included. The most common indication for DC was TBI (86%), followed by ischemic stroke (IS). Eleven studies included TBI patients only. Similarly, most studies reported unilateral hemicraniectomy as the only modality. One study employed bifrontal craniectomy only [65], while seven studies reported bifrontal craniectomy proportions ranging from 5% to 28%. Reported gender distribution was 65% male (early CP 66.4%, late CP 64.2%). There was a significant difference in pooled ages between groups, with late cohorts being younger (early CP 37.2 ± 15 years, late CP 34.2 ± 15.2 years, *p* < 0.0001). Three studies reported a comparison using a cutoff of 45 ± 15 days [7, 25, 61]. These were included in the ultra-early sub-analysis. Additionally, two authors provided data re-stratified by this 45-day cutoff [43, 55].

Six functional neurological scales were used as outcome assessment tools. Ten studies reported more than one functional outcome scale [1, 4, 14, 22, 25, 28, 38, 41, 47, 48, 63]. For pooled analyses, the primary scale was selected; when this could not be identified from the manuscript, the most detailed or comprehensive scale was used instead. MMSE was the only instrument applied for cognitive assessment, although one study also reported the cognitive domain of FIM [22]. Consequently, MMSE was used exclusively for pooled cognitive outcome analysis.

Fourteen studies provided both pre- and post-cranioplasty assessments and were therefore included in baseline and change analyses. Six studies reported post-CP outcomes only. In one study [19], change summary statistics could only be retrieved from a prior meta-analysis [10]. Accordingly, fourteen studies were included in the pre-CP analysis, twenty in the post-CP analysis, and fifteen in the change analysis. Follow-up assessments ranged from less than three days to one year after CP. Four studies reported multiple follow-up assessments [8, 25, 48, 63], of which the latest was used for analysis.

### Overall effect of cranioplasty

**Fig. 2:**
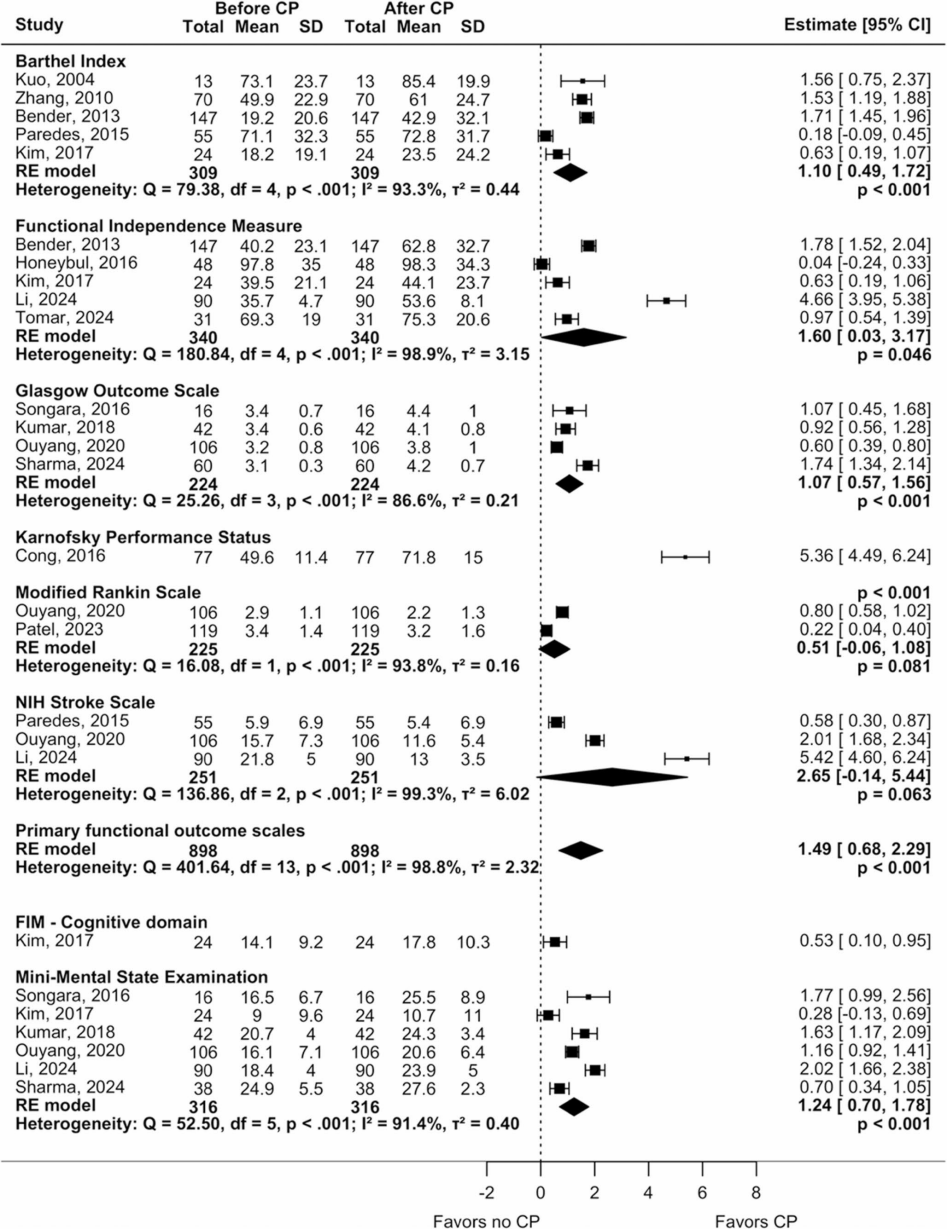
Forest plot of the overall effect of CP on neurological outcomes, irrespective of timing. Effect estimates are presented as standardized mean change coefficients (SMCC), with positive values indicating improvement following cranioplasty. mRS and NIHSS scores inverted, so that positive SMD indicates better function

Fourteen studies reported pre- and post-CP assessments, allowing for analysis of CP effect regardless of timing (Fig. 2). All studies reported increase in scores across all outcome scales. Pooled analysis showed large effect sizes for BI (SMCC = 1.1 [0.49–1.72], I² = 93.3%), FIM (SMCC = 1.6 [0.03–3.17], I² = 98.9%), GOS (SMCC = 1.07 [0.57–1.56], I² = 86.6%), the overall primary functional estimate (SMCC = 1.49 [0.68–2.29], I² = 98.8%), and cognitive outcomes measured by MMSE (SMCC = 1.24 [0.7–1.78], I² = 0%). Heterogeneity was considerable for all pooled analyses.

### Baseline assessment

Fourteen studies reported pre-cranioplasty assessment. Only one study demonstrated a significant baseline difference favoring the early cranioplasty group [25]. In the pooled analysis, no significant differences were observed between early and late cranioplasty groups for any individual scale or for the overall pooled estimate based on primary functional outcomes (SMD = 0.13 [-0.22–0.49]). Heterogeneity was substantial for BI (I^2^ = 59.7%) and considerable for GOS (I^2^ = 92.9%) and the pooled primary functional estimate (I^2^ = 83.4%)

### Post-cranioplasty assessment

**Fig. 3:**
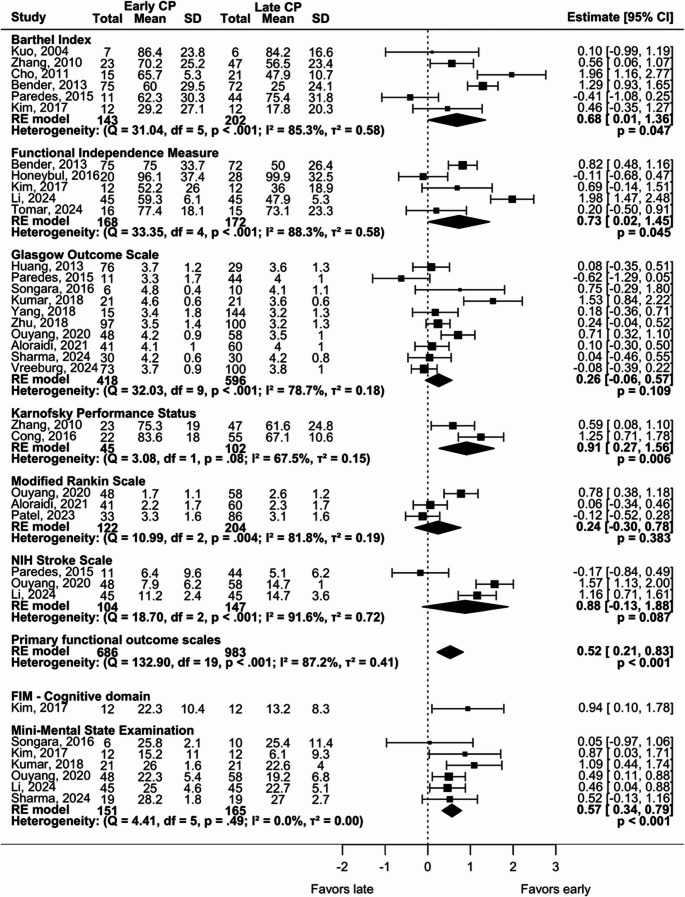
Forest plot of post-cranioplasty neurological outcome scores comparing early versus late CP. Effect estimates are presented as standardized mean differences (SMD), with positive values indicating improved outcome scores in the early cranioplasty group. mRS and NIHSS scores inverted, so that positive SMD indicates better function

**Fig. 4:**
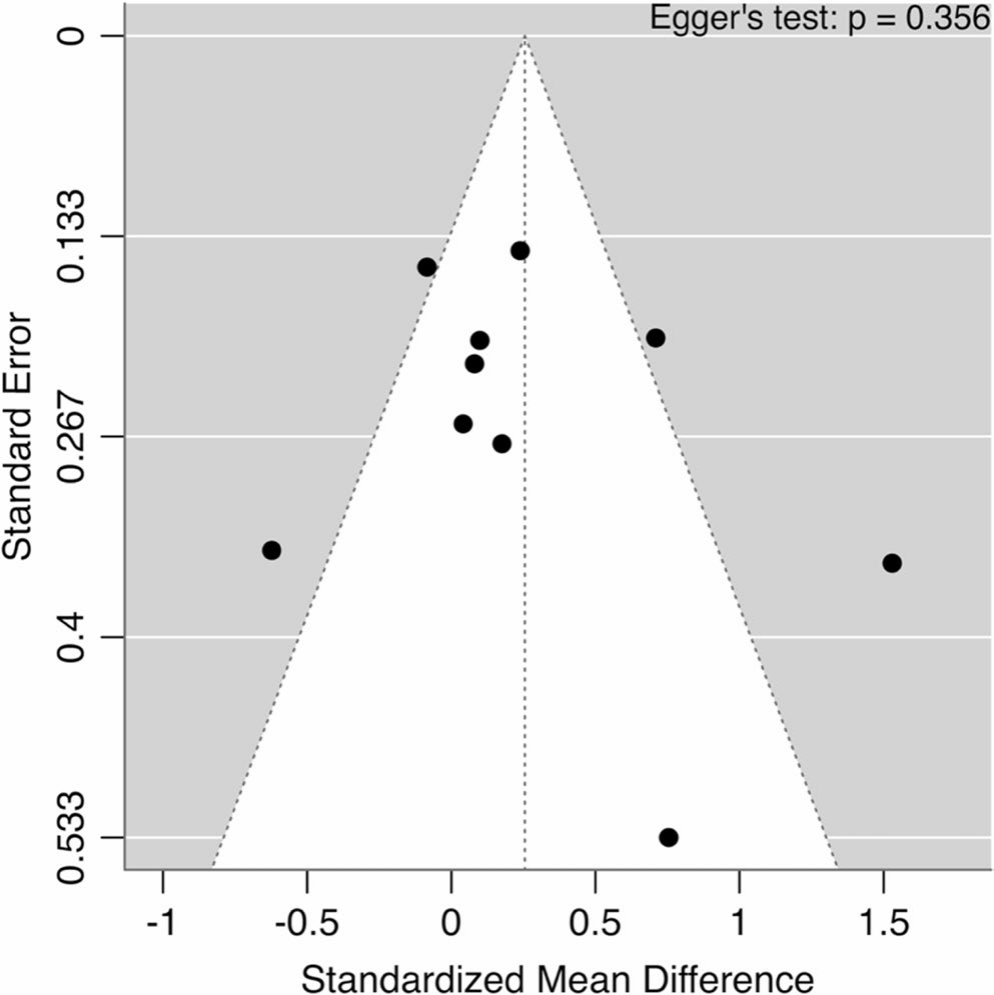
Funnel plot with Egger’s test for post-cranioplasty GOS reports

Twenty studies reported post-cranioplasty assessments (Fig. 3). On pooled analysis, significant differences favoring early cranioplasty were observed for the BI (6 studies, SMD = 0.68 [0.01–1.36], I² = 85.3%), FIM (5 studies, SMD = 0.73 [0.01–1.36], I² = 88.3%), KPS (2 studies, SMD = 0.91 [0.27–1.56], I² = 67.5%), the overall primary functional estimate (SMD = 0.52 [0.21–0.83], I² = 87.2%), and cognitive outcomes measured by MMSE (6 studies, SMD = 0.57 [0.34–0.79], I² = 0%). Pooled estimates also favored early cranioplasty, though without reaching statistical significance, for the GOS (10 studies, SMD = 0.26 [− 0.06–0.57], I² = 78.7%), mRS (3 studies, SMD = 0.24 [− 0.30–0.78], I² = 81.8%), and NIHSS (3 studies, SMD = 0.88 [− 0.13–1.88], I² = 91.6%). Heterogeneity was substantial to considerable across all analyses except for MMSE, which showed complete consistency (I² = 0%). Only the GOS analysis included enough studies for funnel plot inspection and Egger’s test, which did not suggest significant asymmetry (*p* = 0.36, Fig. 4).

### Change in neurological scores in early vs. late cranioplasty

**Fig. 5:**
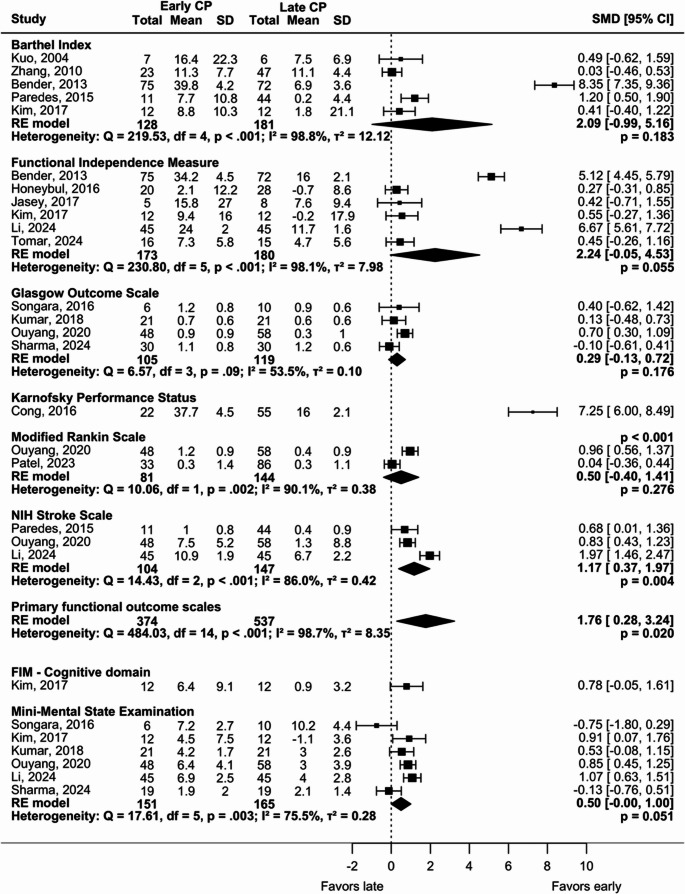
Forest plot of change in neurological outcome scores comparing early versus late CP. Effect estimates are presented as standardized mean differences (SMD) of change in scores, with positive values indicating greater improvement in the early cranioplasty group. mRS and NIHSS scores inverted, so that positive SMD indicates better function

Change in functional or neurological scores was available for 15 studies comparing early and late CP (Fig. 5). In pooled analyses, significant differences favoring early cranioplasty were found for the NIHSS (3 studies, SMD = 1.17 [0.37–1.97], I² = 86%) and for the overall pooled primary functional estimate (SMD = 1.76 [0.28–3.24], I² = 98.7%). Non-significant pooled effects in favor of early cranioplasty were observed for the BI (5 studies, SMD = 2.09 [− 0.99–5.16], I² = 98.8%), FIM (6 studies, SMD = 2.24 [− 0.05–4.53], I² = 98.1%), GOS (4 studies, SMD = 0.29 [− 0.13–0.71], I² = 53.6%), mRS (2 studies, SMD = 0.50 [− 0.40–1.41], I² = 90.1%), and MMSE (6 studies, SMD = 0.50 [− 0.001–1.00], I² = 75.5%). Heterogeneity was substantial to considerable across analyses.

### Subgroup analysis: TBI

**Fig. 6:**
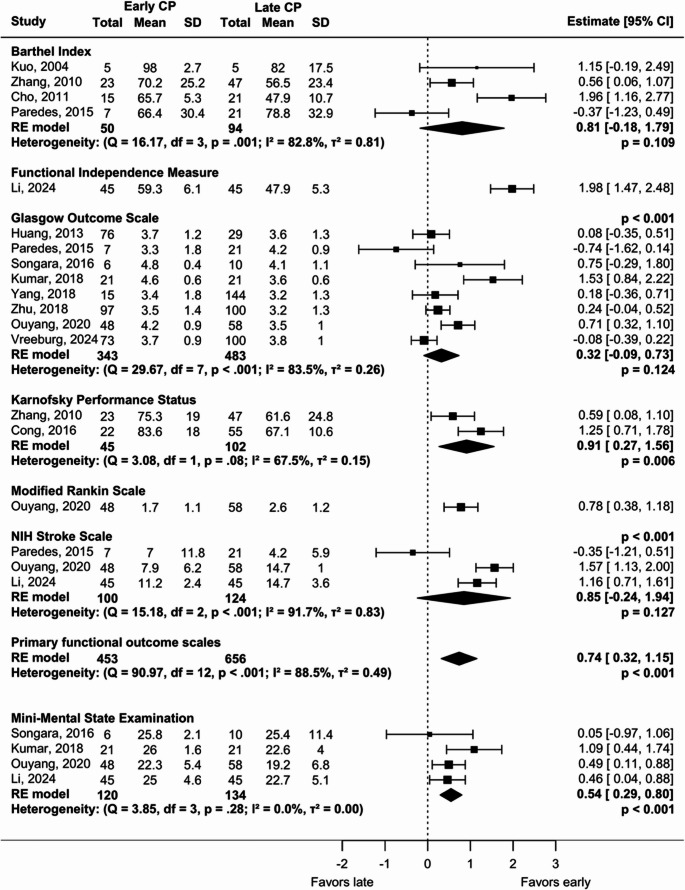
Forest plot of post-CP neurological outcome scores in the TBI subgroup, comparing early versus late CP. Effect estimates are presented as standardized mean differences (SMD), with positive values indicating greater scores in the early cranioplasty group. mRS and NIHSS scores inverted, so that positive SMD indicates better function

**Fig. 7:**
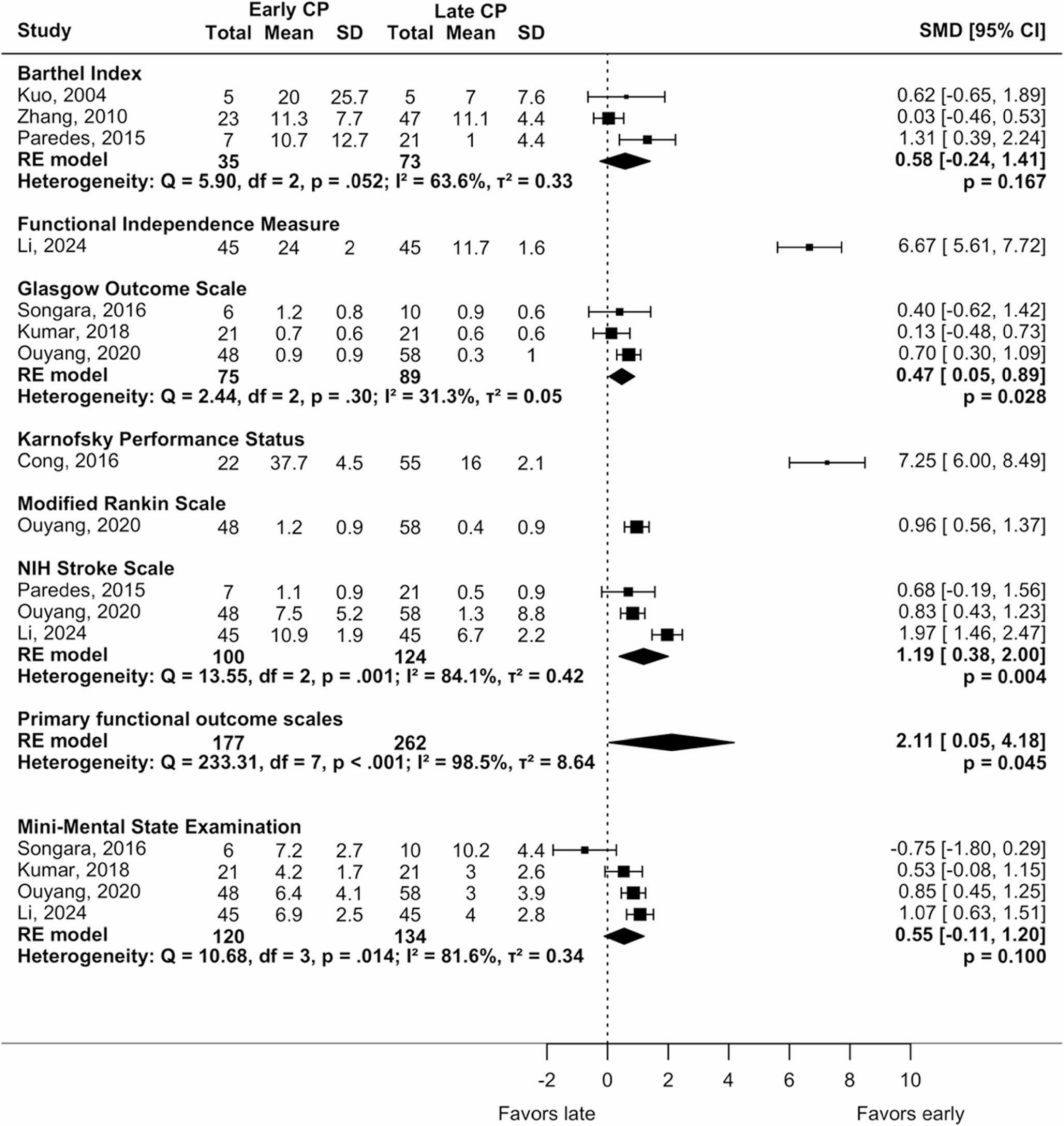
Forest plot comparing change in neurological outcome scores between early and late CP in the TBI subgroup. Effect estimates are presented as standardized mean differences (SMD) of change in scores, with positive values indicating greater improvement in the early CP group. mRS and NIHSS scores inverted, so that positive SMD indicates better function

Eleven studies included TBI patients only, and two additional studies provided sufficient data to reconstruct TBI-specific summary statistics [26, 41]. The pooled primary functional estimate for post-cranioplasty scores remained significant in favor of early cranioplasty (13 studies, SMD = 0.74 [0.32–1.15], I² = 88.5%, Fig. 6). The pooled estimate for change scores was also significant (8 studies, SMD = 2.11 [0.05–4.18], I² = 98.5%, Fig. 7).

### Subgroup analysis: ultra-early cranioplasty

**Fig. 8:**
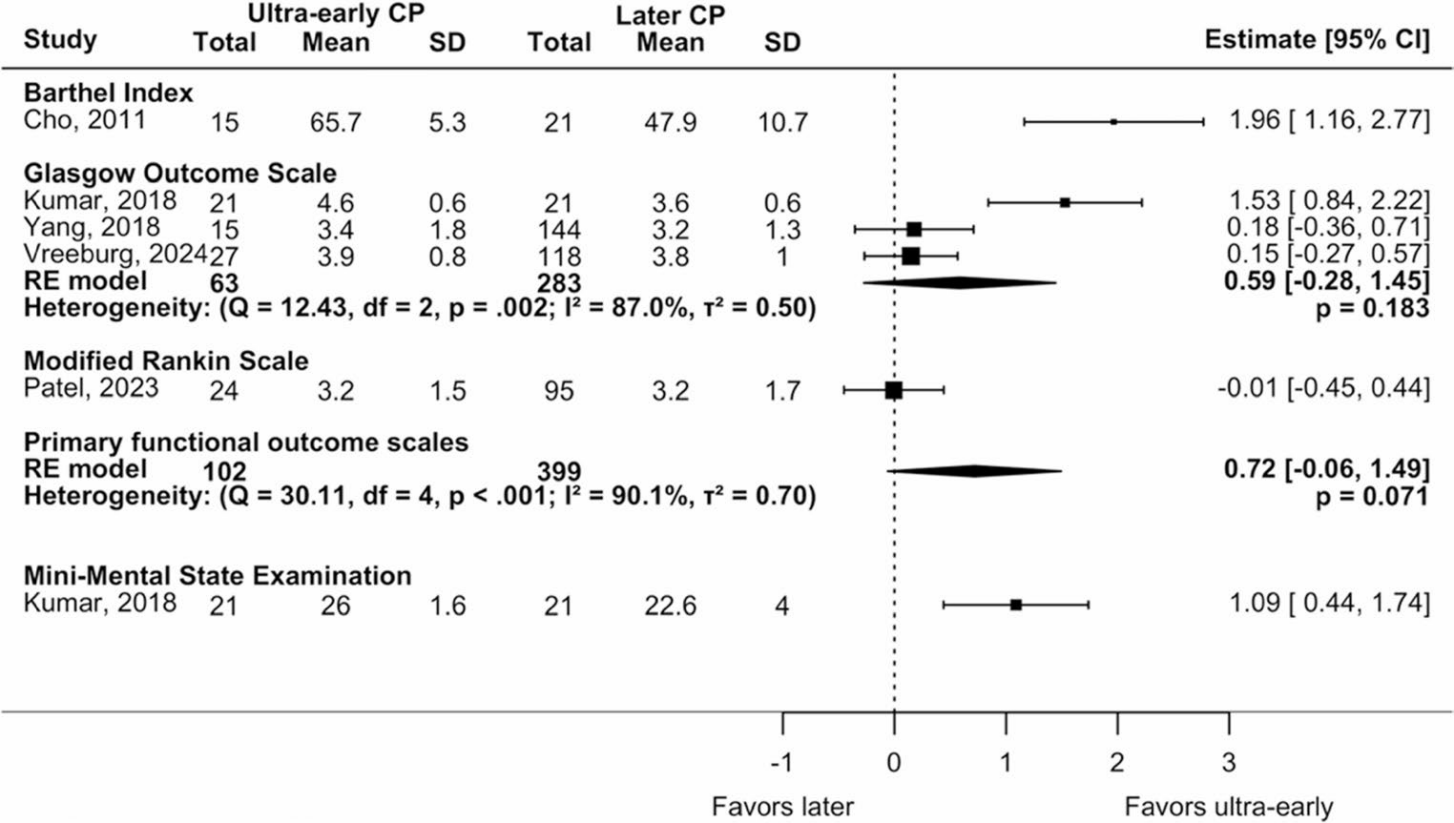
Forest plot of post-CP neurological outcome scores comparing ultra-early versus later CP. Effect estimates are presented as standardized mean differences (SMD), with positive values indicating greater scores in the ultra-early CP group. mRS scores inverted, so that positive SMD indicates better function

**Fig. 9:**
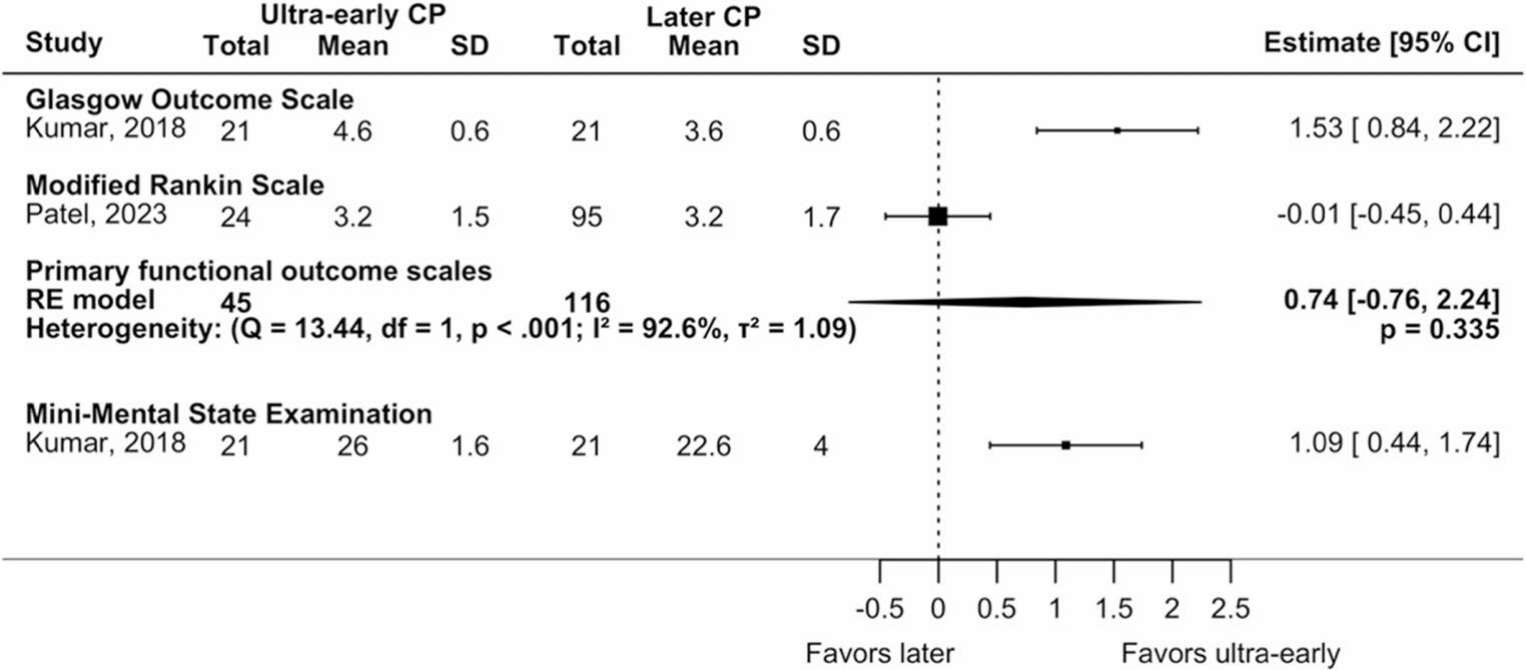
Forest plot comparing change in neurological outcome scores between ultra-early and later. Effect estimates are presented as standardized mean differences (SMD) of change in scores, with positive values indicating greater improvement in the ultra-early CP group. mRS scores inverted, so that positive SMD indicates better function

Post-cranioplasty scores were available from five studies evaluating ultra-early cranioplasty (< 45 days). Scale-specific pooled analysis was feasible for the GOS, showing a non-significant advantage of ultra-early over later cranioplasty (3 studies, SMD = 0.59 [− 0.28–1.45], I² = 87%, Fig. 8). When all five studies were pooled, the overall estimate similarly favored ultra-early cranioplasty without reaching statistical significance (SMD = 0.72 [− 0.06–1.49], I² = 90.1%). Only two studies provided change-score data for this subgroup [25, 43], yielding a non-significant pooled effect (SMD = 0.73 [− 0.76–2.24], I² = 92.6%, Fig. 9).

## Discussion

The present systematic review and meta-analysis provide further support for prior syntheses suggesting that cranioplasty after decompressive craniectomy is associated with improved neurological function, and that earlier cranioplasty may be associated with better recovery than delayed reconstruction [10, 31]. The association was more extensively shown across a wide range of outcome measures, including cognitive assessment tools such as the MMSE. This signal remained in the TBI-only subgroup analysis, while ultra-early CP (within 45 days) did not appear to confer an additional benefit. However, these findings should be interpreted cautiously. The available evidence remains almost entirely observational, with limited adjustment for confounding and only two matched cohort studies. In addition, early and late CP groups are inherently different in clinical practice, as timing is influenced by factors such as medical stability, residual brain swelling, complication burden, and rehabilitation status. Accordingly, the present results support an association rather than a definitive causal effect of earlier timing on neurological recovery. The overall level of evidence remains OCEBM level 3 [37].

The included studies had populations of either exclusively or predominantly TBI patients. We considered DC indication a potentially important confounder in the study results and therefore sought to isolate the effect of cranioplasty in this population. Unfortunately, this could not be explored in other DC indications: only one study, not included in the quantitative synthesis, explored this in malignant infarction patients and found a benefit in earlier cranioplasty for this particular population [60]. Consequently, the present findings apply primarily to the TBI population.

Most included studies involved unilateral hemicraniectomy. Only one study included a bifrontal DC population with bone window collapse [65]. Their reported neurological recovery, as assessed by GOS, demonstrated a benefit of early cranioplasty for patients with a preoperative Glasgow Coma Scale score of 9–12, and the overall effect favored early CP but was not significant. While the overall pooled effect in our meta-analysis favored early reconstruction, subgroup analysis by DC location was not feasible. The low proportion of bifrontal DC patients reflects current practice trends and likewise affects the validity of the findings.

Six functional outcome assessment tools were included. These were grouped according to their prevalence of use and their ability to evaluate overall neurological function and independence. However, the scales differ considerably in range, granularity, and original purpose. BI, FIM, and KPS are more granular measures of function and independence, whereas GOS and mRS are coarser ordinal scales with different ranges, ceiling effects, and clinical interpretations. NIHSS, although widely used, is primarily a deficit scale rather than a global functional measure. The pooled “functional” estimate should therefore be interpreted cautiously as a generalized signal of neurological and functional recovery rather than a precise estimate for any single clinical domain. Notably, the most consistent statistically significant associations were observed in the more granular scales, whereas the coarser ordinal scales often showed directionally similar but non-significant effects. The predominant cognitive assessment tool was MMSE, typically as an adjunct to the previously mentioned functional scales and might help address their limitations.

We observed that cranioplasty, irrespective of timing, was consistently associated with significant functional and cognitive improvement, in agreement with the findings of Malcolm et al. [31]. Significant gains were seen across several scales, including BI, FIM, GOS, and MMSE. However, these findings should not be interpreted as isolating the effect of cranioplasty itself, since recovery after decompressive craniectomy also reflects spontaneous neurological improvement, rehabilitation exposure, and the broader clinical course. Thus, cranioplasty may be one component of recovery rather than its sole determinant.

We note a consistent association between earlier cranioplasty and better post-cranioplasty functional outcomes across multiple measures. This was reflected both in pooled post-cranioplasty comparisons and, more variably, in change-score analyses. At the same time, these findings must be interpreted in light of baseline and selection differences between groups. Although pooled pre-cranioplasty analyses did not show a significant overall difference, this does not establish true clinical comparability, particularly because several studies lacked pre-cranioplasty data and many relevant confounders were not adequately captured. Notably, younger patients were more frequently represented in the delayed group, a pattern that likely reflects clinical selection rather than a true prognostic advantage and may indicate unmeasured differences in injury burden or recovery readiness. In this context, the two matched cohort studies included in the review are especially informative. Zhang et al. matched groups on age, sex, preoperative GCS score, and skull-defect area and still reported better ADL and KPS outcomes in the early group, although interpretation is tempered by differences related to duraplasty status [63]. Zhu et al. likewise used strict matching and found that the apparent benefit of earlier cranioplasty was not uniform across all patients, but concentrated in selected subgroups [65]. Taken together, these data support a possible benefit of earlier reconstruction, while also underscoring the importance of residual confounding and patient selection.

These findings are consistent with the hypothesis that earlier restoration of the cranial vault may favor neurological recovery, potentially through improvements in cerebral perfusion, CSF hydrodynamics, and the physiological environment for rehabilitation and neuroplasticity [29, 46, 49]. These mechanisms are biologically plausible but should not be used to overstate causal inference from the present analysis. Rather, they provide a rationale for why earlier reconstruction may be beneficial in appropriately selected patients.

These findings should also be interpreted alongside the safety literature, which has been more extensively addressed in recent complication-focused reviews [6, 15, 32, 40, 58]. Overall, these reviews do not suggest a consistent increase in overall complications with earlier cranioplasty, although some have reported a higher risk of hydrocephalus [32, 40, 58], whereas others did not find this association to be statistically significant [6, 15, 35]. Considered jointly, the available literature suggests that earlier cranioplasty may be associated with neurological benefit without a clear overall complication penalty, but both efficacy and safety remain influenced by patient selection, study design, and timing definitions.

The association between earlier cranioplasty and better outcomes remained present when analyses were restricted to patients with traumatic brain injury, the most common indication for decompressive craniectomy in the included literature. This consistency reduces, but does not eliminate, concern that the overall findings are driven mainly by diagnostic heterogeneity. Because data for non-TBI indications were limited, however, the present findings should still be interpreted primarily within the context of TBI populations.

In contrast, the ultra-early subgroup (< 45 days) showed a nonsignificant trend toward improved outcomes compared with later reconstruction. Interpretation is limited by the small number of studies and substantial heterogeneity. Moreover, timing definitions varied by up to two weeks around the 45-day threshold, complicating comparisons. Nonetheless, the existing literature hints at a potentially favorable profile for ultra-early intervention: Palavani et al. reported decreased odds of subdural effusion with CP < 35 days [40], while Chasles and Hou, using the 45-day cutoff, observed comparable or lower complication rates [6, 15]. Although underpowered, these studies raise the intriguing possibility that very early reconstruction may reduce complications in addition to improving recovery. This must be explored in future prospective trials.

The rationale for using a 90-day cutoff to define early versus late cranioplasty warrants comment. This threshold was selected because it is the most commonly used binary definition in the literature and facilitates comparison across studies and prior reviews [33]. Nevertheless, it remains partly conventional and may oversimplify what is likely a continuous relationship between timing and recovery. Some studies have evaluated timing beyond a binary framework: Honeybul et al. included the interval between decompression and cranioplasty as a continuous variable in regression modeling of postoperative FIM scores [14], Cong et al. described a reciprocal relationship between time to cranioplasty and postoperative KPS improvement [8], and Kuo et al. reported a negative correlation between interval to reconstruction and neurological improvement after cranioplasty [26]. These observations suggest that future studies may benefit from analyzing time-to-cranioplasty as a continuous exposure rather than relying exclusively on dichotomous thresholds.

## Limitations

The observational nature of the evidence remains the most important limitation. Most studies were retrospective with incomplete adjustment for confounding, although study quality has improved recently with publication of large prospective registries [5, 55]. One report of a randomized trial directly compared early (1–3 months) versus late (6–12 months) titanium-mesh cranioplasty and reported significantly higher BI scores at 30 days in the early group; however, numerical data were unavailable for inclusion in our synthesis [62]. Three ongoing randomized trials may further strengthen the evidence base [34, 42, 59]. High heterogeneity across most analyses also limits inference. The most evident potential source was the wide variability in assessment times after CP, but differences patient selection, outcome definitions, surgical protocols, and reconstructive materials were also likely contributors. The latter may be particularly relevant, as included studies variably used autologous bone, titanium mesh, PMMA, and, in more recent practice, patient-specific implants. These differences may influence complication risk, fit, and perioperative course, and likely contribute to between-study variability even if they could not be formally stratified in the present review. Additional limitations include incomplete availability of primary summary statistics for some studies, imputation of change-score variances, and reliance on MMSE as the dominant cognitive outcome measure, which limits conclusions regarding broader cognitive recovery.

Future research should prioritize well-designed prospective multicenter trials incorporating standardized timing definitions, unified outcome measures, and extended follow-up to evaluate both neurological and quality-of-life outcomes. Integration of perfusion imaging and neurophysiological monitoring could further elucidate mechanisms underlying functional improvement. Development of predictive models incorporating patient-specific factors, such as etiology, defect size, and complication risk, may enable individualized timing strategies.

## Conclusion

Cranioplasty after decompressive craniectomy is associated with significant functional and cognitive improvement. Early cranioplasty, performed within three months, is associated with greater recovery compared with delayed reconstruction, and this effect is directionally consistent across functional scales and within TBI-specific subgroups. However, evidence remains observational, and the association remains uncertain in magnitude with significant heterogeneity and potential confounding. Well-designed multicenter randomized trials are needed to establish the optimal timing threshold that maximizes neurological recovery while ensuring procedural safety.

## Supplementary Information

Supplementary Material 1.

## Data Availability

Data supporting the findings of the study are available on request from the corresponding author.
