# Supplementary material for: Timing of cranioplasty after decompressive craniectomy and neurological recovery: A systematic review and meta-analysis
Source: Neurosurg Rev. 2026 Jun 9;49(1):440. doi: 10.1007/s10143-026-04361-3 (PMC13246589; doi:10.1007/s10143-026-04361-3)
Supplement: Supplementary file 1 — Supplementary Material 1. [file 10143_2026_4361_MOESM1_ESM.docx]

**Supplementary Table 1** Newcastle-Ottawa Scale Assesment

| **Study** | **S1** | **S2** | **S3** | **S4** | **C1** | **C2** | **O1** | **O2** | **O3** | **Total** |
| --- | --- | --- | --- | --- | --- | --- | --- | --- | --- | --- |
| Kuo 2004 | 1 | 1 | 1 | 1 | 0 | 0 | 1 | 1 | 1 | 7 |
| Zhang 2010 | 1 | 1 | 1 | 1 | 1 | 1 | 1 | 1 | 0 | 8 |
| Cho 2011 | 1 | 1 | 1 | 0 | 0 | 0 | 1 | 1 | 1 | 6 |
| Bender 2013 | 1 | 1 | 1 | 1 | 1 | 0 | 1 | 1 | 1 | 8 |
| Huang 2013 | 1 | 1 | 1 | 0 | 0 | 0 | 1 | 1 | 1 | 6 |
| Paredes 2015 | 1 | 1 | 1 | 1 | 0 | 0 | 1 | 0 | 1 | 7 |
| Cong 2016 | 1 | 1 | 1 | 1 | 0 | 0 | 1 | 1 | 1 | 7 |
| Honeybul 2016 | 1 | 1 | 1 | 1 | 0 | 0 | 1 | 0 | 1 | 6 |
| Songara 2016 | 1 | 1 | 1 | 1 | 0 | 0 | 1 | 1 | 0 | 6 |
| Kim 2017 | 1 | 1 | 1 | 1 | 0 | 0 | 1 | 0 | 0 | 5 |
| Jasey 2017 | 1 | 1 | 1 | 1 | 1 | 0 | 1 | 1 | 1 | 8 |
| Yang 2018 | 1 | 1 | 1 | 1 | 0 | 0 | 1 | 1 | 1 | 7 |
| Zhu 2018 | 1 | 1 | 1 | 1 | 1 | 1 | 1 | 1 | 0 | 7 |
| Kumar 2018 | 1 | 1 | 1 | 1 | 0 | 0 | 1 | 1 | 1 | 7 |
| Ouyang 2020 | 1 | 1 | 1 | 1 | 0 | 0 | 1 | 1 | 1 | 7 |
| Aloraidi 2021 | 1 | 1 | 1 | 0 | 0 | 0 | 1 | 0 | 1 | 5 |
| Patel 2023 | 1 | 1 | 1 | 1 | 1 | 0 | 1 | 1 | 1 | 8 |
| Tomar 2024 | 1 | 1 | 1 | 1 | 0 | 0 | 1 | 1 | 1 | 7 |
| Sharma 2024 | 1 | 1 | 1 | 1 | 0 | 0 | 1 | 1 | 1 | 7 |
| Li 2024 | 1 | 1 | 1 | 1 | 1 | 0 | 1 | 1 | 0 | 7 |
| Vreeburg 2024 | 1 | 1 | 1 | 1 | 1 | 1 | 1 | 1 | 0 | 8 |

**S1:** Representativeness of cohort

**S2:** Selection of comparison group

**S3:** Ascertainment of exposure

**S4:** Outcome not present at baseline

**C1–C2:** Comparability (confounder control / matching)

**O1:** Outcome assessment

**O2:** Follow-up duration adequate

**O3:** Adequacy of follow-up

**Supplementary Table 2** Scale selection rationale for pooling

| **Study** | **Eligible reported scales** | **Primary scale for pooled functional analysis** | **Reason for selection** |
| --- | --- | --- | --- |
| Kuo 2004 | BI | **BI** | Only functional scale reported |
| Zhang 2010 | BI, KPS | **BI** | Primary assesment tool of study, with both pre- and post CP assesment;KPS only assessesed 6 months post-CP |
| Cho 2011 | BI | **BI** | Only functional scale reported |
| Bender 2013 | BI, FIM, CRS | **BI** | Primary assessment tool of study |
| Huang 2013 | GOS | **GOS** | Only functional scale reported |
| Paredes 2015 | BI, GOS, NIHSS | **BI** | NIHSS not optimal for functional assesment, BI more granular than GOS |
| Cong 2016 | KPS | **KPS** | Only functional scale reported |
| Honeybul 2016 | FIM, Cognistat | **FIM** | Data taken from previous synthesis, where Cognistat was not reported |
| Songara 2016 | GOS, GCS, MMSE | **GOS** | GOS is the relevant functional outcome; GCS not a recovery scale |
| Kim 2017 | K-MBI, FIM, FIM cognitive domain, K-MMSE, | **BI** | Primary functional assesment tool |
| Jasey 2017 | FIM | **FIM** | Only functional scale reported |
| Yang 2018 | GOS | **GOS** | Only functional scale reported |
| Zhu 2018 | GOS | **GOS** | Only functional scale reported |
| Kumar 2018 | GOSE, MMSE | **GOSE collapsed to GOS** | Collapsed for comparability |
| Ouyang 2020 | GOS, mRS, NIHSS, MMSE | **GOS** | Primary assesment tool of study |
| Aloraidi 2021 | GOS, mRS | **GOS** | Primary assesment tool of study |
| Patel 2023 | mRS | **mRS** | Only functional scale reported |
| Tomar 2024 | FIM | **FIM** | Only functional scale reported |
| Sharma 2024 | GOS, MMSE | **GOS** | Only functional scale reported |
| Li 2024 | FIM, NIHSS, MMSE, NCSE | **FIM** | NIHSS not optimal for functional assesment |
| Vreeburg 2024 | GOSE, QOLIBRI | **GOSE collapsed to GOS** | Only functional scale reported |
